# Biochemical Profiles in Hypertension: Ferritin, Antioxidant Enzymes, and Mineral Levels in Blood Serum

**DOI:** 10.7759/cureus.88338

**Published:** 2025-07-19

**Authors:** Mohamed Salem, James Robenson

**Affiliations:** 1 Biomedical Sciences, Penn State Health Milton S. Hershey Medical Center, Hershey, USA; 2 Neurosurgery, AdventHealth Orlando, Orlando, USA

**Keywords:** antioxidant enzymes, ferritin levels, hypertension, oxidative stress, serum biomarkers

## Abstract

The aim of this study was to evaluate the level of ferritin and some biochemical indices in patients with hypertension. The total number of samples taken was 90, including 60 from hypertensive patients (Group 1: 30 female patients and 30 male patients) aged between 25 and 55 years, and another 30 specimens from healthy individuals (Group 2: 15 female patients and 15 male patients). Samples were obtained from an open-access repository that aggregates information from multiple sources. Specific collection locations were not disclosed to ensure confidentiality and generalizability of the findings. This study assessed concentrations of ferritin, cholesterol, catalase, malondialdehyde (MDA), peroxidase, nitrite, sodium, and potassium in the blood serum. The results showed that Group 1 has higher levels of serum ferritin than the control group, which are significantly different among male patients, but no significant difference between male and female subjects in the control group. Findings revealed that Group 1 had lower cholesterol levels compared to those without high blood pressure. Catalase levels did not differ significantly between groups, although they were found to be higher in male patients of Group 1. Malondialdehyde level was significantly higher in Group 1 as compared to the control group, while peroxidase activity showed no significant difference between groups. Sodium concentration was significantly greater for Group 1, whereas potassium concentration was the highest in the control group. This demonstrates how these biomarkers could help understand hypertension more clearly and suggests potential research areas requiring closer clinical attention.

## Introduction

According to 2015 global estimates, approximately 900 million people were affected by hypertension, making it one of the most prevalent chronic conditions worldwide [1-3]. The condition forces the heart to pump blood through narrowed arteries, increasing vascular resistance. Over time, this strain can lead to arterial wall thickening, atherosclerosis, and eventual weakening of cardiac muscle tissue [4,5]. Hypertension is associated with a wide range of complications, including cardiovascular disease, nephropathy, retinopathy, and coronary artery disease. Ferritin is a cellular iron storage protein composed of two subunits, ferritin heavy chain (FHC) and ferritin light chain (FLC), which form a nanocage structure with 12 nm external diameter and 8 nm inner diameter, capable of sequestering up to 5,000 iron atoms [1]. Beyond its role in iron homeostasis, elevated ferritin levels have been linked to systemic inflammation and increased risk of hypertension, particularly among male patients. In this study, ferritin is examined both as a biomarker of systemic oxidative stress and a potential mechanistic contributor to vascular dysfunction in hypertensive individuals. Additionally, the study evaluates specific antioxidants (e.g., catalase, glutathione, peroxidase, and malondialdehyde (MDA)) and minerals (e.g., sodium and potassium), which are involved in oxidative stress regulation and electrolyte balance, two key factors implicated in hypertension pathophysiology. Oxidative stress in this context is explored primarily as a consequence of hypertension, although its bidirectional role is acknowledged. Therefore, this study aims to assess the relationship between serum ferritin, antioxidant enzyme activity, and mineral levels in hypertensive versus normotensive individuals, with the objective of identifying clinically relevant biomarkers and potential therapeutic targets for improved blood pressure management.

## Materials and methods

Data collection

The study involved 90 samples of hypertensive patients and non-hypertensive individuals. Ages ranged from 25 to 55 years. The samples were divided into specific groups to enable comparison among them. In the control group, which consisted of healthy subjects, there were 30 (15 male patients and 15 female patients) samples [6]. Group 1 was made up of 60 samples of hypertensive patients (30 male patients and 30 female patients). This group was further divided into two subgroups: subgroup C1 comprised 30 hypertensive male patients under antihypertensive treatment, while subgroup C2 included 30 hypertensive male patients who were not receiving treatment. (Note: female patients in Group 1 were analyzed separately and not part of these subgroups.) This subdivision enabled a more detailed examination of how different biochemical markers are influenced by medication status [7].

The enzyme-linked immunosorbent assay (ELISA) technique was employed in determining the concentration of ferritin protein in serum. This is among the most sensitive and specific procedures and follows a set of instructions given by manufacturers, thereby making sure that measurements were precise and dependable. All assays were performed in duplicate to ensure measurement reliability.

Under the already established methodologies, this investigation aimed at measuring the amounts of a number of antioxidants. Cholesterol levels were measured using the Sedlak and Tietz method, a technique widely accepted in clinical biochemistry. The activity of catalase, an important antioxidant enzyme, was evaluated using standard spectrophotometric methods consistent with those applied by Simic et al. [8]. Measurement of malondialdehyde (MDA) was done according to Guidet’s procedure since it is an indicator of lipid peroxidation and oxidative stress [9,10]. The Vanuffelen approach was employed to examine the level of peroxidase activity in controlling cells against oxidative damage [11,12]. Also, using the Hillman method, we determined nitrite concentration that reflects nitric oxide (NO) levels and endothelial function [13]. These various techniques offered a comprehensive analysis of antioxidant defenses within the study population.

Measurement of mineral levels

Spectrum Company, Spain, used automated devices in measuring sodium and potassium, minerals necessary to maintain the balance of body electrolytes [14,15]. The testing followed Guder’s procedure, which guarantees accuracy and uniformity of findings for these specific minerals. In hypertensive patients, these mineral levels are important for assessing possible electrolyte imbalance [16,17]. Reference ranges for serum sodium (135-145 mmol/L) and potassium (3.5-5.0 mmol/L) were used to contextualize abnormal findings.

Statistical analysis

Statistical analysis was performed using the Statistical Package for the Social Sciences (SPSS) version 26.0 (IBM Corp., Armonk, NY). The purpose of this analysis was to find out the significance of differences between hypertensive patients and healthy controls. Normality tests were performed prior to comparisons, and independent samples t-tests were used for group comparisons. Any p-value smaller than 0.05 was considered to have statistical significance, thus showing that the differences observed were not random in nature. No adjustments were made for multiple comparisons. Missing data were minimal; any incomplete records were excluded pairwise from the relevant statistical analyses. This strict statistical methodology enabled the research findings to be valid and reliable.

Disclaimer

This study utilized a pre-collected dataset obtained from multiple sources and posted on an open repository. The dataset was anonymized and did not contain any personally identifiable information. As the data collection was conducted by multiple anonymous sources, there was no direct interaction with human subjects by the authors. Consequently, no explicit institutional review board (IRB) approval was obtained for this study. The authors have adhered to all ethical standards and guidelines for secondary data analysis, ensuring that the use of the dataset complies with relevant ethical and legal requirements.

## Results

The research outcomes indicated that hypertensive patients have differing levels of ferritin, antioxidant enzyme activities, and minerals compared to healthy controls. These discrepancies highlight the complex biochemical alterations associated with hypertension. In hypertensive patients, elevated ferritin levels suggest increased iron deposition and potential oxidative stress, while altered levels of antioxidant enzymes such as catalase and glutathione indicate a disruption in redox homeostasis. Variations in sodium and potassium concentrations reflect disturbances in electrolyte regulation commonly linked to hypertensive pathology. Notably, the potassium levels reported for hypertensive groups (e.g., 12.533 mmol/L in G1 male patients and 14.649 mmol/L in G2 female patients) exceed physiological norms (typically 3.5-5.0 mmol/L), which may reflect either data entry or unit conversion errors and warrant further verification. Additionally, “peroxy nitrate” values were reported, although this may refer to peroxynitrite; clarification is needed regarding the precise oxidative marker measured.

Group 1 (G1: hypertensive male and female patients) was compared to Group 2 (G2: control male and female patients) across all biochemical parameters. Sex-based comparisons within the hypertensive group (G1 male patients versus G1 female patients) were also analyzed, although no statistically significant differences were observed. Independent samples t-tests were used to compare means across groups, as shown in Table 1 and Table 2. All continuous variables were tested for normality using the Shapiro-Wilk test before analysis; data met assumptions for parametric testing. These findings underscore the importance of tracking specific biomarkers, particularly ferritin, MDA, and serum ions, in managing hypertension and identifying therapeutic targets.

**Table 1:** Mean ± SD for various parameters with significant differences indicated Similar letters indicate no significant differences, while different letters indicate significant differences. C1: control group for male patients, G1: hypertensive male patient group, C2: control group for female patients, G2: hypertensive female patient group, SD: standard deviation, GSH: glutathione, MDA: malondialdehyde

Parameters	C1 (male) (mean ± SD)	G1 (male) (mean ± SD)	C2 (female) (mean ± SD)	G2 (female) (mean ± SD)
Ferritin (ng/mL)	27.656 ± 8.334^b^	110.650 ± 38.387^a^	27.857 ± 5.313^b^	95.653 ± 47.820^a^
GSH (μmol/L)	8.828 ± 0.936^a^	3.760 ± 0.945^b^	8.391 ± 0.857^a^	3.587 ± 0.802^b^
Catalase (IU/L)	0.687 ± 0.044^a^	0.365 ± 0.065^b^	0.685 ± 0.044^a^	0.470 ± 0.057^b^
MDA (μmol/L)	29.910 ± 5.611^c^	87.306 ± 33.436^a^	29.805 ± 5.742^c^	74.448 ± 11.130^b^
Peroxy nitrate (μmol/L)	25.520 ± 3.240^c^	41.863 ± 1.713^b^	25.734 ± 3.267^c^	52.955 ± 2.441^a^
K (mmol/L)	3.467 ± 0.127^c^	12.533 ± 2.401^b^	3.584 ± 0.287^c^	14.649 ± 2.368^a^
Na (mmol/L)	135.263 ± 2.921^a^	107.105 ± 13.531^b^	136.210 ± 2.149^a^	104.111 ± 26.649^b^

**Table 2:** Ferritin level in the blood serum of the samples under study according to gender C1: control group for male patients, G1: hypertensive male patient group, C2: control group for female patients, G2: hypertensive female patient group

Group	Ferritin (ng/mL)
C1	27.656
G1	110.65
C2	27.857
G2	95.653

Protein ferritin

Ferritin levels were significantly higher in hypertensive patients (G1) than in normotensive controls (G2), as shown in Table 2. Among male patients, mean serum ferritin was 110.650 ± 38.387 ng/mL in G1 versus 27.656 ± 8.334 ng/mL in G2. Similarly, G1 female patients had a mean ferritin level of 95.653 ± 47.820 ng/mL compared to 27.857 ± 5.313 ng/mL in G2 female patients. While both male and female hypertensive patients showed marked increases compared to their respective controls, sex-based comparisons within G1 did not reveal statistically significant differences. These elevations may reflect both increased iron storage and oxidative stress activity in hypertensive individuals.

Glutathione levels

One of the most prominent non-enzymatic antioxidants responsible for scavenging free radicals is reduced glutathione (GSH), which, when depleted, results in decreased quantities in the bloodstream. This study measured only reduced GSH; oxidized glutathione (GSSG) was not separately quantified, and total glutathione (GSH + GSSG) was not calculated. Glutathione disulfide (GSSG), the oxidized form, results from the dimerization of two GSH molecules and serves as a useful indicator of oxidative stress within cells and tissues [15]. Free radicals such as hydrogen peroxide rapidly oxidize GSH [18], so decreased GSH and increased GSSG levels are often correlated with severe oxidative stress [19]. Cell membranes are damaged by reactive species such as hydroxyl radicals (OH), which contribute to oxidative stress through degradation of membrane proteins and lipids [20]. This oxidative burden also inhibits nitric oxide function, which plays a key role in maintaining vascular endothelial tone. Reduced nitric oxide activity leads to arterial stiffness, contributing to vascular disease and hypertension, which are associated with many cardiovascular risk factors [21-23].

Stress conditions such as hypertension, hyperlipidemia, and atherogenesis are tightly linked to oxidative stress, inflammation, and macrophage activation, all of which contribute to elevated blood pressure [24-26]. The relationship between oxidative stress and blood pressure has been supported by multiple studies. Elevated oxidative markers have been observed in hypertensive populations, particularly among the elderly [27,28].

In this study, GSH levels were significantly lower in hypertensive groups compared to normotensive controls, as shown in Table 3. Among male patients, Group C1 (controls) had a mean GSH level of 8.328 ± 0.936 μmol/L, while Group G1 (hypertensive male patients) had 3.760 ± 0.945 μmol/L (p < 0.001, independent samples t-test). Similarly, among female patients, GSH levels declined from 8.391 ± 0.857 μmol/L in Group C2 to 3.587 ± 0.802 μmol/L in Group G2 (p < 0.001). No adjustments were made for confounding variables such as age, body mass index (BMI), or medication use due to dataset limitations.

**Table 3:** Glutathione levels in the blood serum of the samples according to gender C1: control group for male patients, G1: hypertensive male patient group, C2: control group for female patients, G2: hypertensive female patient group, GSH: glutathione

Group	GSH (µmol/L)
C1	8.328
G1	3.76
C2	8.391
G2	3.587

Catalase enzyme activity in blood serum

Our findings are consistent with Pantopoulos (2018) [29], who also reported decreased catalase enzyme activity in the plasma of patients with various degrees of essential hypertension. This reduction in catalase activity is due to its depletion in neutralizing free radicals and their toxic derivatives, such as hydrogen peroxide and superoxide anion, within the cell. Furthermore, an increase in the lipoprotein/high-density lipoprotein ratio and thus low-density lipoprotein cholesterol levels occurs as a result of tissue damage from increased consumption rate of antioxidants during cases of oxidative stress.

Mean catalase enzyme activity was significantly different between male patients (0.365 ± 0.065 units/L) and female patients (0.687 ± 0.044 units/L) in group G1, as shown in Table 4. On the other hand, for group G2, male patients showed higher catalase activity compared to female patients (male patients: 0.685 ± 0.044 units/L; female patients: 0.470 ± 0.057 units/L). Overall, there was a decrease in the mean levels of catalase enzyme, irrespective of gender, with no significant differences between male and female patients, as shown in Table 4.

**Table 4:** Activity of catalase enzyme in the blood serum of the samples according to gender C1: control group for male patients, G1: hypertensive male patient group, C2: control group for female patients, G2: hypertensive female patient group

Group	Catalase (IU/L)
C1	0.687
G1	0.365
C2	0.685
G2	0.470

MDA levels in blood serum

Table 1 also shows that the mean malondialdehyde (MDA) level for hypertensive male patients (Group G1) was 87.306 ± 33.436 μmol/L, compared to 29.910 ± 5.611 μmol/L in control male patients (Group C1), reflecting a 191.9% relative increase and an absolute difference of 57.396 μmol/L (p < 0.001). Similarly, hypertensive female patients (G2) had an MDA level of 74.448 ± 11.130 μmol/L, while control female patients (C2) had 29.805 ± 5.742 μmol/L, indicating a 149.7% increase (p < 0.001) (Table 5). These elevated MDA values may appear unusually high and warrant verification; however, the results are consistent with data derived from the applied colorimetric assay (based on thiobarbituric acid-reactive substances). No significant gender differences were observed within the hypertensive group (G1 versus G2). Age and antihypertensive treatment status were not controlled for in this analysis, which may have influenced oxidative stress levels.

**Table 5:** Malondialdehyde levels in the blood serum of the samples according to gender C1: control group for male patients, G1: hypertensive male patient group, C2: control group for female patients, G2: hypertensive female patient group, MDA: malondialdehyde

Group	MDA (µmol/L)
C1	29.91
G1	87.306
C2	29.805
G2	74.448

Regarding peroxynitrite, the values reported (e.g., 41.863 ± 1.713 μmol/L in G1 male patients) were measured using a biochemical assay labeled “peroxy nitrate.” It is unclear whether this reflects direct quantification of peroxynitrite or surrogate detection via nitrate/nitrite levels, as the dataset did not specify the method. However, the sharp increase compared to control male patients (25.520 ± 3.240 μmol/L; p < 0.001) suggests significant oxidative activity. Female hypertensives (G2) had even higher values at 52.955 ± 2.441 μmol/L, compared to 25.734 ± 3.267 μmol/L in C2 female patients (p < 0.001). These results further support elevated oxidative stress among hypertensive patients, although potential confounders, such as medication use and age, were not adjusted for due to dataset limitations.

Blood serum pyruvate levels

For male patients, Table 1 indicates an average pyruvate level at 41.863 ± 1.713 μmol/L, while in the control group G1, it was at 25.520 ± 3.240 μmol/L. Female patients averaged at 52.955 ± 2.441 μmol/L, while in the control group, it was at 25.734 ± 3.267 μmol/L. This showed a significant increase in pyruvate levels in the blood serum of male and female patients as compared to the control group, but no significant difference between the male and female groups was observed. The reason for elevated peroxynitrite levels is the increased production of free radicals, in particular superoxide ions and nitric oxide, which interact with cellular constituents, causing cellular damage. In this case, there is an increase in intracellular peroxynitrite production in the cell sap, leading to oxidative stress. This is clearly supported by the data presented in Table 6. 

**Table 6:** Peroxynitrite levels in the blood serum of the samples according to gender C1: control group for male patients, G1: hypertensive male patient group, C2: control group for female patients, G2: hypertensive female patient group

Group	Peroxy nitrate (µmol/L)
C1	25.52
G1	41.863
C2	25.734
G2	52.955

Blood serum sodium levels

Table 1 shows that hypertensive male patients (G1) had an average serum sodium level of 107.105 ± 13.531 mmol/L, significantly lower than control male patients (C1), who had 135.263 ± 2.921 mmol/L (p < 0.001). Similarly, hypertensive female patients (G2) had an average sodium concentration of 104.111 ± 26.649 mmol/L, compared to 136.210 ± 2.149 mmol/L in control female patients (C2) (p < 0.001) (Table 7). While sodium levels were significantly lower in hypertensive individuals compared to controls, no statistically significant sex-based differences were observed within the hypertensive or control groups.

**Table 7:** Sodium levels in the blood serum of the samples according to gender C1: control group for male patients, G1: hypertensive male patient group, C2: control group for female patients, G2: hypertensive female patient group

Group	Na (mmol/L)
C1	135.263
G1	107.105
C2	136.21
G2	104.111

Sodium and potassium concentrations were measured using automated biochemical analyzers provided by Spectrum Company (Spain), following Guder’s protocol, which includes standard calibration with internal controls for clinical electrolyte measurements [14,15]. However, the potassium values reported for hypertensive groups (12.533 ± 2.401 mmol/L in G1 male patients and 14.649 ± 2.368 mmol/L in G2 female patients) are above normal physiological reference ranges (3.5-5.0 mmol/L) and may reflect either unit conversion errors, undetected hemolysis, or data entry issues. These values should be revalidated or interpreted with caution.

No exclusion criteria were applied regarding conditions that may affect electrolyte balance, such as chronic kidney disease, endocrine disorders, or diuretic use, due to the retrospective and anonymized nature of the dataset. Additionally, electrolyte values were not adjusted for fasting status or time of day, which may have introduced biological variability. These limitations should be considered when interpreting the abnormal potassium concentrations reported in hypertensive subjects.

Blood serum potassium level

Male patients were shown to have an average potassium level of 12.533 ± 2.401 mmol/L, while that for the control group G1 was 3.467 ± 0.127 mmol/L, as demonstrated in Table 1. Female patients had 14.649 ± 2.368 mmol/L against 3.584 ± 0.287 mmol/L for the control group. This indicates a significant rise in blood serum potassium levels of both male and female groups, unlike the differences between male and female participants observed in Table 8.

**Table 8:** Potassium levels in the blood serum of the samples according to gender C1: control group for male patients, G1: hypertensive male patient group, C2: control group for female patients, G2: hypertensive female patient group

Group	K (mmol/L)
C1	3.467
G1	12.533
C2	3.584
G2	14.649

## Discussion

Ferritin, an intracellular protein that stores and releases iron, has been implicated in the pathophysiology of hypertension. Elevated serum ferritin levels have been associated with increased blood pressure, particularly among male patients [11,12,22]. Our study confirmed that hypertensive male patients (G1) exhibited significantly higher ferritin levels (110.650 ± 38.387 ng/mL) than their normotensive counterparts (C1: 27.656 ± 8.334 ng/mL; p < 0.001). Similarly, hypertensive female patients (G2: 95.653 ± 47.820 ng/mL) had higher ferritin levels than control female patients (C2: 27.857 ± 5.313 ng/mL; p < 0.001). However, we found no significant sex-based differences in ferritin levels within the hypertensive group itself (G1 versus G2), contradicting earlier narrative mentions of gender-based variation. The previously reported values of 3.760 ± 0.945 μg/L and 3.587 ± 0.802 μg/L refer instead to glutathione, not ferritin, and were mistakenly attributed.

Ferritin’s role in hypertension likely stems not only from its iron-regulating function but also from its behavior as an acute-phase reactant and inflammatory mediator [23-26]. Elevated ferritin levels may reflect systemic inflammation, oxidative stress, or both, each contributing to vascular dysfunction and hypertension [27,28]. It is also elevated in metabolic syndrome, obesity, and insulin resistance [29]. Given that ferritin is nonspecific and reactive to multiple physiological stressors, its use as a diagnostic marker for chronic disease must be interpreted cautiously, especially since hypertensive patients are more likely than the general population to suffer from acute inflammatory or infectious comorbidities.

Our findings also confirmed significantly elevated malondialdehyde (MDA) levels in hypertensive patients, a key marker of lipid peroxidation. G1 male patients had an MDA level of 87.306 ± 33.436 μmol/L versus 29.910 ± 5.611 μmol/L in C1 controls (p < 0.001), while G2 female patients recorded 74.448 ± 11.130 μmol/L compared to 29.805 ± 5.742 μmol/L in C2 (p < 0.001). While these values appear elevated, they are consistent with levels reported in clinical studies using thiobarbituric acid-reactive substance assays. No adjustment was made for age or medication status, which could influence oxidative stress levels. Similarly, peroxynitrite (reported as “peroxy nitrate”) was significantly higher in hypertensive groups, although it remains unclear whether it was directly measured or inferred via nitrite/nitrate assays. Clarifying the exact biochemical technique used for peroxynitrite detection would be necessary for full interpretability.

The study also observed substantial changes in serum electrolytes. Sodium levels were significantly reduced in hypertensive groups compared to controls (G1: 107.105 ± 13.531 mmol/L versus C1: 135.263 ± 2.921 mmol/L; G2: 104.111 ± 26.649 mmol/L versus C2: 136.210 ± 2.149 mmol/L; p < 0.001 for both). However, the potassium values reported (12.533 ± 2.401 mmol/L for G1 male patients and 14.649 ± 2.368 mmol/L for G2 female patients) are biologically implausible, far exceeding the normal reference range (3.5-5.0 mmol/L). These values may reflect data entry errors, improper sample handling, or unit mislabeling. No exclusion criteria were applied for conditions such as renal impairment or diuretic use, which could confound sodium/potassium balance, and electrolyte values were not corrected for fasting status or time of day, both of which influence serum levels.

Prior literature already establishes the role of sodium in hypertension pathogenesis, particularly through increased water retention and vascular tone modulation [30]. While our findings are consistent with this body of work, they do not provide new mechanistic insight. What they do reinforce is the clinical importance of monitoring electrolyte status in hypertensive patients, particularly in those receiving pharmacologic intervention. Future studies should stratify patients by medication class (e.g., angiotensin-converting enzyme (ACE) inhibitors and diuretics) to better delineate drug-related effects on electrolyte and oxidative markers.

The use of pre-collected open-access data enabled broader sample access but introduces variability in sampling protocols, measurement instruments, and clinical documentation. As a result, external validity and data reliability are limited. The lack of control over patient comorbidities, dietary factors, or timing of sample collection further restricts interpretation. Future research should involve larger, prospectively designed studies that account for confounders such as age, BMI, medication use, renal function, and dietary intake to establish causal relationships.

Practical implications and future directions

The biomarkers examined, particularly ferritin, MDA, and antioxidant enzyme levels, have potential utility in stratifying hypertensive patients by oxidative risk and tailoring antioxidant-based therapeutic interventions. There is growing interest in adjunct therapies aimed at restoring redox balance, including dietary antioxidants, iron modulation, and NO-enhancing agents. Our results support the rationale for testing such interventions in high-ferritin hypertensive populations. However, validation in larger and longitudinal cohorts is required to assess their predictive power and clinical utility. Existing large-scale studies (e.g., National Health and Nutrition Examination Survey (NHANES)) could provide a platform for further correlation analysis.

## Conclusions

Hypertensive patients, both treated and untreated, demonstrated significantly altered levels of several biochemical markers compared to normotensive controls. Serum ferritin, malondialdehyde (MDA), and peroxynitrite were consistently elevated, indicating heightened oxidative stress and disrupted iron metabolism. Although glutathione and catalase levels were also altered, these changes were not uniformly significant across subgroups. These results align with previous research suggesting oxidative imbalance as a contributor to hypertension pathogenesis. However, observed potassium elevations in hypertensive individuals exceeded physiological norms and may reflect measurement inconsistencies or data integrity concerns.

Sodium levels were significantly lower in hypertensive patients, but conclusions are limited by the lack of exclusion criteria for renal disease, medication use, or other confounders. The use of a pre-collected open-access dataset enhanced accessibility but limited control over essential clinical variables, reducing generalizability and precluding causal inference. Despite these limitations, ferritin and MDA emerged as promising biomarkers with potential applications in hypertension risk stratification. Prior studies have identified ferritin as a cardiovascular risk predictor and MDA as a marker of oxidative injury. Future research should validate these markers in larger, stratified populations and explore their therapeutic relevance in clinical settings.
